# Supplementary material for: Psychometric properties and factorial structure of the Spanish version of the HLS-EU-Q16 questionary in Venezuelan adults
Source: PLoS One. 2025 Jun 25;20(6):e0324782. doi: 10.1371/journal.pone.0324782 (PMC12193711; doi:10.1371/journal.pone.0324782)
Supplement: S1 Appendix — (DOCX) [file pone.0324782.s001.docx]

**HEALTH LITERACY SCALE**

For the following questions, on a scale of 1 to 5, with 1 being “very easy” and 5 being “very difficult” indicate the degree of difficulty you would find in performing the following activities:

| Actividades | 1 | 2 | 3 | 4 | 5 |
| --- | --- | --- | --- | --- | --- |
| 1. Find information about the treatments your doctor prescribes for your medical conditions. |  |  |  |  |  |
| 1. Find out where to get professional help when you are sick (e.g., doctor, pharmacist, psychologist). |  |  |  |  |  |
| 1. Understanding what the doctor says |  |  |  |  |  |
| 1. Understand the doctor's or pharmacist's instructions on how to take prescribed medications. |  |  |  |  |  |
| 1. Assess when you may need a second opinion from another physician. |  |  |  |  |  |
| 1. Use the information your doctor gives you to help you make decisions about your illness. |  |  |  |  |  |
| 1. Follow your doctor's or pharmacist's instructions |  |  |  |  |  |
| 1. Find information on how to deal with mental health problems such as stress or depression. |  |  |  |  |  |
| 1. Understand health warnings related to habits such as smoking, low physical exercise or excessive alcohol consumption. |  |  |  |  |  |
| 1. Understand why you need to have early disease screening or medical check-ups (e.g., mammogram, blood sugar and blood pressure test). |  |  |  |  |  |
| 1. Evaluate the reliability of health risk information appearing in the media (e.g., television, Internet, or other information media). |  |  |  |  |  |
| 1. Decide how to protect themselves from disease based on information provided by the media (e.g., newspapers, brochures, Internet and other information media). |  |  |  |  |  |
| 1. Find activities that are good for your mental well-being (e.g., meditation, exercise, walking, pilates, etc.). |  |  |  |  |  |
| 1. Understand the health advice given by family and friends. |  |  |  |  |  |
| 1. 15. Understand information provided by the media on how to improve their health (e.g., Internet, newspapers, magazines). |  |  |  |  |  |
| 1. Evaluate which of your daily habits affect your health (e.g., habits related to alcohol consumption, eating habits, exercise, etc.). |  |  |  |  |  |

**HLS-EU-Q16 Spanish version applied to the Venezuelan adult population**

Para las siguientes preguntas, en una escala del 1 al 5, siendo 1 “muy difícil” y 5 “muy fácil” indique cuál es el grado de dificultad que encontraría para realizar las siguientes actividades:

| Actividades | 1 | 2 | 3 | 4 | 5 |
| --- | --- | --- | --- | --- | --- |
| 1. Encontrar información sobre los tratamientos que le indica el médico para sus enfermedades |  |  |  |  |  |
| 1. Averiguar donde conseguir ayuda profesional cuando se encuentra enfermo (p. ejemplo, médico, farmacéutico, psicólogo) |  |  |  |  |  |
| 1. Entender lo que dice el médico |  |  |  |  |  |
| 1. Entender las instrucciones del médico o farmacéutico sobre cómo tomar las medicinas recetadas |  |  |  |  |  |
| 1. Valorar cuándo puede necesitar una segunda opinión de otro médico |  |  |  |  |  |
| 1. Utilizar la información proporcionada por el médico para tomar decisiones sobre su enfermedad |  |  |  |  |  |
| 1. Seguir las instrucciones de su médico o farmacéutico |  |  |  |  |  |
| 1. Encontrar información sobre la manera de abordar problemas de salud mental como el estrés o la depresión |  |  |  |  |  |
| 1. Comprender las advertencias sanitarias relacionadas con hábitos como fumar, hacer poco ejercicio físico o beber alcohol en exceso |  |  |  |  |  |
| 1. Comprender por qué necesita hacerse pruebas de detección precoz de enfermedades o chequeos médicos (p. ejemplo, mamografía, prueba de azúcar en sangre y presión arterial) |  |  |  |  |  |
| 1. Evaluar la confiabilidad de la información sobre riesgos para la salud que aparece en los medios de comunicación (p. ejemplo, televisión, Internet u otros medios de información) |  |  |  |  |  |
| 1. Decidir cómo protegerse de las enfermedades gracias a la información que proporcionan los medios de comunicación (p. ejemplo, periódicos, folletos, Internet y otros medios de información) |  |  |  |  |  |
| 1. Encontrar actividades que sean buenas para su bienestar mental (p. ejemplo, meditación, ejercicio, paseos, pilates, etc |  |  |  |  |  |
| 1. Comprender los consejos sobre salud que dan la familia y los amigos |  |  |  |  |  |
| 1. Comprender la información proporcionada por los medios de comunicación sobre cómo mejorar su salud (p. ejemplo, Internet, periódicos, revistas) |  |  |  |  |  |
| 1. Evaluar cuáles de sus hábitos diarios afectan a su salud (p. ejemplo, costumbres relacionadas con el consumo de alcohol, hábitos alimenticios, ejercicios, etc.) |  |  |  |  |  |
