# Supplementary material for: Psychometric properties and factorial structure of the Spanish version of the HLS-EU-Q16 questionary in Venezuelan adults
Source: PLoS One. 2025 Jun 25;20(6):e0324782. doi: 10.1371/journal.pone.0324782 (PMC12193711; doi:10.1371/journal.pone.0324782)
Supplement: Attachment — Submitted filename: Response to Reviewers (17feb).docx [file pone.0324782.s004.docx]

**Response to Reviewers**

Psychometric properties and factorial structure of the Spanish version of the HLS-EU-Q16 questionary in Venezuelan adults.

The authors wish to sincerely thank the reviewers for their valuable comments and contributions, which have significantly improved the quality of this scientific article.

| **Observación del Revisor** | **Respuesta y Modificación Realizada** |
| --- | --- |
| Add more literature in the introduction, cite more than one research for the factors thought to be associated with Health literacy (if possible include various socioeconomic locations and global and regional data.) | References were added that relate LH to chronic disease management, the role of caregivers in diabetes, the influence of education and financial deprivation on LH levels, as well as its impact on prevention and health promotion in cancer patients, adolescents and in the context of COVID-19. Studies linking LH to self-care, perioperative risk reduction and cognitive impairment were also included. |
| Line 132 Find a better term for 'Accidental sampling', additionally it's not clear where exactly the sample population was gotten from (at their homes, public areas, schools?) | The term “Accidental sampling” was replaced by “Convenience sampling”, as this term is more accurate and widely accepted in the methodological literature to describe a non-probability sampling based on the accessibility and willingness of the participants.  In addition, it was specified in the manuscript that the data was collected through electronic surveys (Google Forms) and face-to-face interviews in public institutions, parks or shopping malls. These locations were strategically selected to ensure a diverse representation of the population, covering different socioeconomic and educational levels. |
| Generally, can you further explain the difference between assessing the psychometric qualities of the scale VS validating the tool in local context | The distinction between the assessment of psychometric properties (reliability, validity and factor structure analysis) and the validation of the tool in the local context (linguistic and cultural adaptation) has been added to the manuscript. This improves the methodological clarity of the study. |
| The adaptation of the HLS-EU-Q16 is mentioned briefly, and many researcher are unaware of this tool. Please expand on its items. | In response to the reviewer’s comment, we have expanded the introduction to detail the linguistic and cultural adaptation of the HLS-EU-Q16. We incorporated references to previous adaptations in different cultural contexts (Gústafsdóttir et al., 2020; Storms et al., 2017) to emphasize the importance of maintaining the instrument’s validity. Additionally, we explicitly describe our process, including translation, back-translation, and cognitive interviews with 30 participants to ensure its comprehension and relevance in the Venezuelan context.  Examples of some items that make up the questionnaire were also provided. |
| Could you provide more details about the informed consent process and how privacy and confidentiality were ensured? | The description of the informed consent process has been expanded to clarify that participants provided written consent, were informed about the study objectives, and assured confidentiality. It is now explicitly stated that data collection was anonymous, and no personally identifiable information was recorded." |
| Why a factor of 1.5 was used? a reference would be useful. | The use of a factor of 1.5 to account for potential data inaccuracies is not a commonly documented practice in standard psychometric analyses. Therefore, we have removed it from the manuscript to ensure methodological rigor and adherence to established psychometric principles. |
| On what basis did you use these thresholds in the scoring system to determine the categories (inadequate, problematic, and adequate)? | The justification for the categorization thresholds has been expanded in the manuscript by incorporating references to previous studies that have used different classification approaches for health literacy.   - Sørensen et al. (2015) proposed a four-level classification, combining the ‘inadequate’ and ‘problematic’ levels into a single “limited” literacy category for some analyses. - Nolasco et al. (2020) used a simplified two-level classification, distinguishing between ‘inadequate or problematic’ (0-12 points) and ‘sufficient’ (13-16 points). - Other studies, such as García-Vera et al. (2023), Poza Méndez et al. (2023), and Kampouroglou et al. (2021), adopted a three-level categorization, similar to the one used in this study.   Following these methodological precedents, the present study establishes three levels of health literacy: inadequate (16-40 points), problematic (41-60 points), and adequate (61-80 points). The existence of multiple categorization models across different studies demonstrates that classifications are adapted based on research objectives and target populations. The three-level approach chosen for this study provides a more precise differentiation of health literacy levels within the Venezuelan context, facilitating the identification of groups that require greater intervention in terms of access to and comprehension of health information. |
| Why two statistical softwares has been used? please elaborate. | All analyses were performed using Jeffrey’s Amazing Statistics Program (JASP) version 0.18.3.0. Therefore, references to IBM Statistical Package for the Social Sciences (SPSS) version 24 (IBM Inc., Chicago, IL, USA) have been removed to ensure consistency in the description of the statistical software used |
| Any changes to the reference list should be mentioned in the rebuttal letter that accompanies your revised manuscript | To strengthen the introduction and broaden the context of the study, new references addressing the relationship between health literacy and various health conditions were incorporated. The added references include recent studies on the influence of health literacy on the care of people with diabetes (Semerci Çakmak et al., 2025), college education (Dauletkaliyeva et al., 2025), pediatric care (Kampouroglou et al., 2021), gynecological cancer prevention (Aksoy et al., 2025), bladder cancer patient follow-up (Keles et al., 2025), health promotion behaviors in adolescents (You & Ahn, 2025), health literacy during the COVID-19 pandemic (Hou et al., 2025), preoperative assessments (Kim & Sweitzer, 2025), and the relationship between cognitive impairment and health literacy in patients with heart failure (Wu et al., 2025).  Additionally, in response to the reviewer’s comment, we have expanded the introduction to provide a more detailed description of the **linguistic and cultural adaptation of the HLS-EU-Q16.** To highlight the importance of maintaining the instrument’s validity across different populations, we incorporated **Storms et al. (2017),** who suggested modifications to improve clarity and contextual relevance in certain population groups. |
| Please review your reference list to ensure that it is complete and correct. | All references have been carefully reviewed. |
